# Facile and efficient one-pot synthesis of benzimidazoles using lanthanum chloride

**DOI:** 10.1186/2191-2858-3-7

**Published:** 2013-08-06

**Authors:** Yekkirala Venkateswarlu, Sudhagani Ramesh Kumar, Panuganti Leelavathi

**Affiliations:** 1 Department of Chemistry, Osmania University College for Women, Koti, Hyderabad 500095, India

**Keywords:** Benzimidazoles, Aldehydes, *o*-Phenylenediamine, Lanthanum chloride

## Abstract

**Background:**

We report the synthesis of benzimidazoles using lanthanum chloride as an efficient catalyst. One-pot synthesis of 2-substituted benzimidazole derivatives from *o*-phenylenediamine and a variety of aldehydes were developed under mild reaction conditions.

**Results:**

We have examined the effect of different solvents using the same reaction conditions. The yield of the product varied with the nature of the solvents, and better conversion and easy isolation of products were found with acetonitrile. In a similar manner, the reaction with *o*-phenylenediamine and 3,4,5-trimethoxybenzaldehyde was carried out without any solvents. The observation shows that the reaction was not brought into completion, even after starting for a period of 9 h, and the reaction mixture showed a number of spots in thin-layer chromatography.

**Conclusions:**

In conclusion, lanthanum chloride has been employed as a novel and efficient catalyst for the synthesis of benzimidazoles in good yields from *o*-phenylenediamine and a wide variety of aldehydes. All of the reactions were carried out in the presence of lanthanum chloride (10 mol%) in acetonitrile at room temperature.

## Background

**Figure 1:**
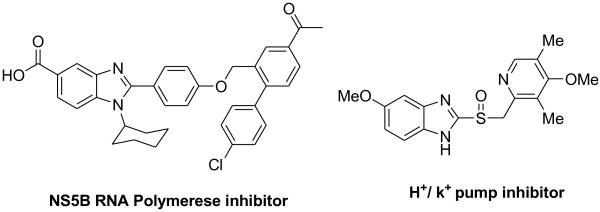
**Scheme of NS5B polymerase and H**^**+**^**/K**^**+ **^**inhibitors.**

Benzimidazole nucleus is found in a variety of naturally occurring compounds such as vitamin B12 and its derivatives; it is structurally similar to purine bases. Benzimidazoles and its derivatives represent one of the most biologically active classes of compounds, possessing a wide spectrum of activities, and these are well documented in the literature. They show selective nonpeptide luteinizing hormone-releasing hormone antagonist, lymphocyte-specific kinase inhibitor, *N*-methyl-d-aspartate antagonist, 5-liopoxygenase inhibitor, NS5B polymerase inhibitor (Figure 1), neuropeptide YY1 receptor antagonist, nonpeptide thrombin inhibitor, γ-aminobutyric acid receptor, factor Xa inhibitor, and poly (ADP-ribose) polymerase inhibitor. DNA-minor groove-binding agents possess antitumor activity, topoisomerase I inhibitors, angiotensin II inhibitors, and proliferation inhibitors. Several benzimidazole derivatives find applications that include antimicrobial, antihypertensive, anticancer antiulcer, antifungal, antihistamine activity, herbicides, and other veterinary applications as promising drugs in different therapeutic categories. The benzimidazole moieties express a significant activity against several viruses such as HIV, herpes (HSV-1), RNA influenza, human cytomegalovirus, selective angiotensin II inhibitors, and 5-HT3 antagonists. In addition, benzimidazoles are very impotent intermediates in synthetic routes and serve as ligands for asymmetric catalysts [1-8]. The high profile of biological applications of the benzimidazole compounds has prompted the emergence of extensive studies of their syntheses. In this context, numerous efforts have been made to synthesize benzimidazole derivatives. One of the most common methods for the preparation of benzimidazole derivatives involves the condensation of an *o*-phenylenediamine and carbonyl compounds such as aldehydes and acid derivatives. The condensation of *o*-phenylenediamine with carboxylic acid often requires strong acidic conditions and high temperatures [9,10]. The other method involves the oxidative cyclodehydrogenation of Schiff bases, which is generated from *o*-phenylenediamine and aldehydes in the presence of various catalysts.

This is the most popular approach in general for the synthesis of benzimidazole derivatives. The catalysts used are CAN, K_3_PO_4_, oxone, sulfamic acid, DDQ, PhI (OAc)_2_, iodine, and KHSO_4_[11-17]. In addition, several catalysts such as metal halides and metal oxychlorides, [18-22] metal oxides, PTSA, metal triflates, air, [23-30] ionic liquid, heteropoly acid, BDSB [31-33], proline, solid-supported catalysts, polymer-supported catalysts [34,35], and microwave-promoted [36-39] and clayzic [40] reactions have been reported in the literature. Unfortunately, many of these methods suffer from drawbacks such as drastic reaction conditions, low yields, tedious workup procedures, and co-occurrence of several side reactions. As a consequence, the introduction of an efficient and mild method is still needed to overcome these limitations.

As part of our research program in developing various synthetic methodologies [41-46], we report the synthesis of benzimidazoles using lanthanum chloride (LaCl_3_) as an efficient catalyst. The catalyst is known as an efficient catalyst in the literature for various organic transformations [47-52].

## Methods

Melting points were recorded on a Buchi R-535 apparatus (BUCHI Labortechnik AG, Flawi, Switzerland) and were uncorrected. Infrared (IR) spectra were recorded on a PerkinElmer FT-IR 240-c spectrophotometer (PerkinElmer Instruments, Branford, CT, USA) using KBr discs. Hydrogen-1 nuclear magnetic resonance (^1^H NMR) spectra were recorded on a Gemini-200 spectrometer (Varian Medical Systems, Palo Alto, CA, USA) in CDCl_3_ using TMS as internal standard. Mass spectra were recorded on a Finnigan MAT 1020 (Thermo Fisher Scientific, Waltham, MA, USA) mass spectrometer operating at 70 eV.

## Results and discussion

**Scheme 1:**
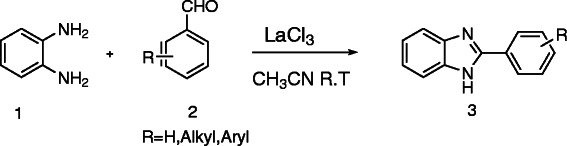
**Reaction between 1,2-phenylenediamine (1) and 3,4,5-trimethoxybenzaldehyde (2) that yielded 2-(3,4,5-trimethoxyphenyl)-1*H*-benzo[d]imidazole (3).**

In a typical experiment, a reaction was made to occur between 1,2-phenylenediamine (OPD) (**1**) and 3,4,5-trimethoxybenzaldehyde (**2**) in the presence of LaCl_3_ in acetonitrile at room temperature to afford the corresponding product, **2**-(3,4,5-trimethoxyphenyl)-1*H*-benzo[d]imidazole (**3**), in excellent yield. The reaction was completed within 2 h (Scheme 1).

We have examined the effect of different solvents using the same reaction conditions, as shown in Table 1. The yield of the product varied with the nature of the solvents; better conversion and easy isolation of products were found with acetonitrile. Acetonitrile dissolves a wide range of ionic and nonpolar compounds. In a similar manner, the reaction with *o*-phenylenediamine and 3,4,5-trimethoxybenzaldehyde was carried out without any solvents. The observation shows that the reaction was not brought into completion, even after starting for a period of 9 h, and the reaction mixture showed a number of spots in thin-layer chromatography (TLC).

**Table 1:** Comparative study of the solvent system

**Number**	**Solvent**	**Time (h)**	**Yield (%)**
1	CH_3_CN	2.0	95
2	CH_3_OH	4.0	80
3	Dioxane	5.0	75
4	THF	6.0	70
5	Toluene	7.0	65
6	DMF	5.0	6
7	DCM	8.0	50
8	-	9.0	-

In a similar manner, a comparative study on the role and requirement of the catalyst for condensation has been carried out, and the obtained results are clearly shown in Table 2. The reactants for this reaction are also *o*-phenylenediamine and 3,4,5-trimethoxybenzaldehyde in acetonitrile. From our observation, a catalytic amount (10 mol%) of LaCl_3_ was enough to complete the conversion of aldehyde and *o*-phenylenediamine into the required condensation product.

**Table 2:** Comparative study of catalyst

**Number**	**Amount of catalyst** (**LaCl**_**3**_) (**eq**)	**Time** (**h**)
1	0.1	2.0
2	0.2	2.0
3	0.4	3.5
4	0.6	3.5
5	0.8	3.0
6	1.0	3.0
7	No catalyst	15

A blank experiment was carried out with *o*-phenylenediamine and 3,4,5-trimethoxybenzaldehyde in the absence of the catalyst LaCl_3_, and the required 3,4,5-trimethoxybenzimidazole product was not found even after stirring for 15 h. Finally, it was decided that the suitable conditions for condensation is in a solvent and in the presence of an activator or promoter. As shown in Table 3, aromatic, heteroaromatic, α-unsaturated and β-unsaturated aldehydes, and aliphatic aldehydes were reacted very well to afford the corresponding products of benzimidazole derivatives in very good to excellent yields. In general, the aromatic aldehydes having electron-donating groups and heteroaromatic compounds are reacting a little faster when compared with other aldehydes. In a similar manner, the aliphatic aldehydes and aromatic aldehydes containing electron-withdrawing groups are reacting comparatively a little slower in terms of conversion as well as yields, benzaldehyde and OPD, in the presence of the catalyst Lacl_3_. In general, all the reactions were completed within 2 to 4 h, and the obtained yields were 85% to 95%.

**Table 3:** Lanthanum chloride-catalyzed synthesis of benzimidazoles

**Entry**	**Diamine**	**Aldehyde**	**Product (3a-3q)**	**Time (h)**	**Yields (%)**
a				2.0	95
b				2.5	88
c				2.0	91
d				2.0	9.0
e				3.0	87
f				2.0	85
g				2.5	92
h				4.0	90
i				3.0	88
j				2.5	86
k				3.0	90
l				2.5	91
m				3.0	87
n				4.0	85
o				3.0	88
p				2.5	86
q				2.5	89

## Experimental

### General procedure

A mixture of *o*-phenylenediamine (1.0 mmol) and aldehyde (1.2 mmol) in the presence of lanthanum chloride (10 mol%) was stirred in acetonitrile (5 ml) at room temperature. The progress of the reaction was monitored by TLC. After completion of the reaction as indicated by TLC, the solvent was removed under reduced pressure. The residue was dissolved in ethyl acetate and washed with water and brine. The organic layer was dried over Na_2_SO_4_ and concentrated under reduced pressure. The crude products were purified by column chromatography. All the products were identified by their ^1^H NMR, IR, and mass spectroscopy data.

### Spectral data for selected compounds

#### ***2-(3,4,5-Trimethoxyphenyl)-1H-benzo[d]imidazole (3a)***

For this compound, the white solid’s melting point was 259°C. The IR (KBr) frequency (*υ*) values were as follows: 2,924, 2,851, 1,601, 1,495, 1,463, 1,416, 1,282, 1,096, 1,020, 899, 801, 749, and 693 cm−^1^. The ^1^H NMR (DMSO-d_6_) chemical shift (*δ*) values were as follows: 3.90 (s, 3H), 4.00 (s, 6H), 7.43 to 7.60 (m, 2H), 7.65 (s, 2H), and 7.85 to 7.95 (m, 2H). The electron ionized mass spectrometry (EIMS) mass-to-ratio (*m*/*z*) values and corresponding percentage were as follows: 285 (m^+1^ 100%), 269 (10%), 255 (10%), and 224 (5%).

#### ***4-(1H-Benzo[d]imidazol-2-yl)-N,N-dimethyl benzenamine (3b)***

For this compound, the white solid’s melting range was 288°C to 290°C. The IR (KBr) *υ* values were as follows: 2,853, 2,800, 1,740, 1,611, 1,561, 15,276, 1,446, 1,389, 1,362, 1,324, 1,278, 1,230, 1,200, 1,167, 1,106, 1,064, 948, 819, 800, 744, 769, and 583 cm^−1^. The ^1^H NMR (DMSO-d_6_) *δ* values were as follows: 2.90 (s, 6H), 6.70 (dd, 2H), 6.95 (d, 2H), 7.10 to 7.25 (m, 2H), and 7.60 (dd, 2H). The EIMS *m/z* values and percentage were as follows: 238 (m^+1^ 100%), 157 (30%), 134 (80%), and 109 (10%).

#### ***2-(4-(Allyoxy)-3-methoxyphenyl)-1H-benzo[d]imidazole (3c)***

For this compound, the solid’s IR (KBr) *υ* values were as follows: 3,063, 2,923, 2,853, 1,886, 1,747, 1,649, 1,607, 1,580, 1,449, 1,460, 1,422, 1,387, 1,316, 1,250, 1,215, 1,180, 1,138, 1,027, 991, 924, 866, 805, 763, 743, 628, and 594 cm^−1^. The ^1^H NMR (DMSO-d_6_) *δ* values were as follows: 3.75 (s, 3H), 4.55 (d, 2H), 5.25 (d, 1H), 5.40 (t, 2H), 5.95 to 6.10 (m, 1H), 6.60 (d, 1H) 6.73 (t, 1H), 7.15 to 7.35 (m, 2H), 7.50 to 7.60 (m, 2H), and 7.80 (d, 2H). The EIMS *m/z* values and corresponding percentage were as follows: 280 (m^+1^ 100%) and 242 (80%).

#### ***2-(Furan-2-yl)-1H-benzo[d]imidazole (3d)***

For this compound, the solid’s melting point was 296°C. The IR (KBr) *υ* values were as follows: 2,927, 2,857, 1,741, 1,609, 1,545, 1,462, 1,379, 1,189, 1,069, 751, and 597 cm^−1^. The ^1^H NMR (DMSO-d_6_) *δ* values were as follows: 6.30 (d, 2H), 7.15 to 7.35 (m, 2H), 7.40 (d, 1H), and 7.65 (d, 2H). The EIMS *m/z* values and corresponding percentage were as follows: 184 (m^+1^ 100%), 158 (20%), 137 (5%), and 133 (5%).

#### ***(E)-2-Styryl-1H-benzo[d]imidazole (3e)***

For this compound, the solid’s melting range was 201°C to 203°C. The IR (KBr) *υ* values were as follows: 3,377, 3,027, 2,924, 2,853, 1,948, 1,805, 1,633, 1,598, 1,495, 1,449, 1,402, 1,355, 1,326, 1,284, 1,194, 1,153, 1,070, 1,018, 963, 918, 841, 737, 691, and 558 cm^−1^. The ^1^H NMR (DMSO-d_6_) *δ* values were as follows: 6.40 (dd, 1H), 6.55 (d, 1H), 7.15 to 7.55 (m, 7H), and 7.70 (d, 2H). The EIMS *m/z* values and corresponding percentage were as follows: 220 (m^+1^ 15%), 195 (5%), 174 (5%), 155 (5%), 144 (5%), and 134 (5%).

#### ***2-(4-Fluorophenyl)-1H-benzo[d]diazole (3f)***

For this compound, the white solid’s melting point was 248°C. The IR (KBr) *υ* values were as follows: 3,053, 2,930, 1,663, 1,624, 1,545, 1,486, 1,440, 1,315, 1,277, 1,229, 1,094, 1,034, 1,004, 972, 833, 795, 746, 690, 618, and 568 cm^−1^. The ^1^H NMR (DMSO-d_6_) *δ* values were as follows: 7.15 to 7.20 (m, 2H), 7.20 to 7.40 (m, 2H), 7.45 to 7.52 (m, 2H), 7.60 to 7.70 (m, 2H), and 8.00 (brs, 1H). The EIMS *m/z* values and corresponding percentage were as follows: 212 (m^+^ 100%), 193 (5%), 215 (15%), 168 (5%), 155 (5%), 136 (5%), 129 (5%), and 95 (5%).

#### ***2-p-Tolyl-1H-benzo[d]imidazole (3g)***

For this compound, the white solid’s melting point was 275°C. The IR (KBr) *υ* values were as follows: 3,397, 3,027, 2,922, 2,858, 1,813, 1,514, 1,481, 1,452, 1,412, 1,383, 1,348, 1,282, 1,250, 1,183, 1,157, 1,114, 1,021, 987, 823, 746, and 612.cm^−1^. The ^1^H NMR (DMSO-d_6_) *δ* values were as follows: 2.35 (s, 3H), 4.42 (brs, 1 NH), 6.95 (d, 2H), 7.10 (d, 2H), 7.28 (d, 2H), and 7.55 (d, 2H). The EIMS *m/z* values and corresponding percentage were as follows: 208 (m^+^ 100%), 195 (15%), 179 (20%), 161 (10%), 153 (10%), 149 (5%), 140 (20%), 136 (5%), 126 (10%), and 122 (5%).

#### ***3-(1H-Benzo[d]imidazol-2-yl)-2-chloro-6-methylquinoline (3h)***

For this compound, the solid’s IR (KBr) *υ* values were as follows: 3,073, 1,585, 1,493, 1,435, 1,392, 1,331, 1,280, 1,227, 1,147, 1,031, 929, 816, 748, 711, 646, 579, and 483 cm^−1^. The ^1^H NMR (DMSO-d_6_) *δ* values were as follows: 2.60 (s, 3H), 7.25 (d, 2H), 7.70 (d, 1H), 7.80 (d, 2H), 7.90 (d, 2H), and 8.80 (s 1H). The EIMS *m*/*z* values and corresponding percentage were as follows: 294 (m^+^ 70%), 290 (10%), 274 (40%), 258 (5%), 246 (5%), 230 (5%), 212 (5%), 191 (10%), and 169 (5%).

#### ***2-Phenyl-1H-benzo[d]imidazole (3i)***

For this compound, the white powder’s melting point was 295°C. The IR (KBr) *υ* values were as follows: 3,406, 3,047, 1,589, 1,540, 1,443, 1,409, 1,483, 1,275, 1,118, 736, and 704 cm^−1^. The ^1^H NMR (DMSO-d_6_) *δ* values were 4.50 (brs, 1H), 7.20 to 7.40 (m, 2H), 7.50 to 7.75 (m, 5H), 7.70 (d, 2H), and 8.25 (d, 2H). EIMS *m*/*z* values and corresponding percentage were as follows: 195 (m^+^ 10%), 175 (5%), and 160 (5%).

#### ***4-(1H-Benzo[d]imidazole-2yl) phenol (3j)***

For this compound, the white powder’s melting range was 229°C to 230°C. The IR (KBr) *υ* values were as follows: 3,376, 3,290, 3,027, 2,807, 1,697, 1,611, 1,591, 1,515, 1,443, 1,394, 1,268, 1,246, 839, and 745 cm^−1^. The ^1^H NMR (DMSO-d_6_) *δ* values were as follows: 6.90 (d, 1H), 7.05 to 7.15 (m, 4H), and 7.75 (d, 2H). The EIMS *m*/*z* values and corresponding percentage were as follows: 210 (m^+^ 100%), 193 (5%), 191 (20%), 183 (10%), 181 (5%), 169 (40%), 154 (5%), 137 (5%).

#### ***2-(4-(Benzyloxy)-3-methoxyphenyl)-1H-benzo[d]imidazole (3k)***

For this compound, the IR (KBr) *υ* values were as follows: 3,036, 2,924, 2,853, 1,738, 1,604, 1,497, 1,458, 1,384, 1,321, 1,240, 1,209, 1,175, 1,132, 1,028, 992, 905, 802, 740, 697, 641, and 572, and 465 cm^−1^. The ^1^H NMR (DMSO-d_6_) *δ* values were as follows: 3.73 (s, 3H), 5.15 (s, 2H), 6.55 (d, 1H), 6.55 (d, 1H), 6.75 (dd, 2H) 7.10 to 7.50 (m, 7H), and 7.80 (d, 2H). The EIMS *m*/*z* (first set) values and corresponding percentage were as follows: 330 (m^+^ 60%), 313 (10%), 305 (20%), 289 (5%), 261 (30%), 245 (20%), 227 (100%), 210 (20%), 201 (50%), 195 (20%), 157 (20%), 100 (30%), 91 (10%), and 89 (5%). The EIMS *m*/*z* (second set) values and corresponding percentage were as follows: 245 (m^+^ 100%), 243 (5%), and 141 (10%). The EIMS *m*/*z* (third set) values and corresponding percentage were as follows: 245 (m^+^ 100%), 243 (5%), and 141 (10%).

#### ***2-(3-Chlorophenyl)-1H-benzo[d]imidazole (31)***

For this compound, the white powder’s melting range was 232°C to 234°C. The IR (KBr) *υ* values were as follows: 3,059, 1,619, 1,593, 1,440, 1,421, 1,269, 836, and 750 cm^−1^. The ^1^H NMR (DMSO-d6 MHz) *δ* values were as follows: 7.45 to 7.60 (m, 4H), 7.62 to 7.72 (m, 2H), and 8.30 to 8.45 (m, 2H). The EIMS *m*/*z* value with its corresponding percentage was 229 (m^+^ 100%).

#### ***2-(Naphthalene-2yl)-1H-benzo[d]imidazole (3m)***

For this compound, the white powder’s melting range was 218°C to 219°C. The IR (KBr) *υ* values were as follows: 3,425, 3,047, 2,924, 2,853, 1,624, 1,605, 1,447, 1,385, and 748 cm^−1^. The ^1^H NMR (DMSO-d6) *δ* values were as follows: 6.70 to 6.90 (m, 2H), 7.20 to 7.35 (m, 2H), 7.55 to 7.80 (m 4H), and 7.90 to 8.10 (m, 2H). The EIMS *m*/*z* values and corresponding percentage were as follows: 245 (m^+^ 100%), 243 (5%), and 141 (10%).

#### ***(E)-2-(Pent-en-2-yl)-1H-benzo[d]imidazole (3n)***

For this compound, the IR (KBr) *υ* values were as follows: 3,064, 2,963, 2,923, and 1,648 cm^−1^. The ^1^H NMR (DMSO-d6) *δ* values were as follows: 1.10 (t, 3H), 1.80 (m, 2H), 1.95 to 2.10 (m, 2H), 5.90 (dd, 1H), 7.30 (d, 2H), and 7.75 (d, 2H). The EIMS *m*/*z* value was 187.

#### ***2-(4-Nitrophenyl)-1H-benzo[d]imidazole (3o)***

For this compound, the yellow powder’s melting point was 314°C. The IR (KBr) *υ* values were 3,042, 1,604, 1,515, 1,434, 1,353, 854, 745, and 710 cm^−1.^. The ^1^H NMR (DMSO-d6) *δ* values were as follows: 7.10 to 7.15 (m, 2H), 7.30 (d, 1H), 7.35 (d, 1H), 7.40 (t, 1H), 7.45 (t, 1H), 8.0 (dd, 2H), and 13.0 (brs, 1H). The EIMS *m*/*z* values and corresponding percentage were as follows: 240 (m^+^ 100%), 226 (5%), 211 (10%), 194 (20%), and 182 (5%).

#### ***2-(Pyridine-2-yl)-1H-benzo[d]imidazole (3p)***

For this compound, the solid’s melting range was 245°C to 248°C. The IR (KBr) *υ* values were as follows: 3,068, 1,449, 1,402, 1,280, and 746 cm^−1^. The ^1^H NMR (DMSO-d6) *δ* values were as follows: 6.85 (m, 2H), 7.00 to 7.10 (m, 1H), 7.45 to 7.55 (m, 1H), 7.80 to 7.90 (m, 2H), 8.10 (t, 1H), and 8.65 (d, 1H). EIMS *m*/*z* value with its corresponding percentage was 196 (m^+^ 15%).

#### ***4-(1H-Benzo[d]imidazole-2yl) benzonitrile (3q)***

For this compound, the white crystal solid’s melting point was 262°C. The IR (KBr) *υ* values were as follows: 3,417, 3,047, 2,912, 2,222, 1,605, 1,454, 1,408, and 748 cm^−1^. The ^1^H NMR (DMSO-d6) *δ* values were as follows: 5.50 (brs, 1H), 7.45 to 7.60 (m, 2H), 7.82 to 7.90 (m, 2H), 8.05 (d, 2H), and 8.50 (d, 2H). The EIMS *m*/*z* values and corresponding percentage were as follows: 220 (m^+^ 100%), 211(10%), 196 (5%), and 186 (5%).

## Conclusions

In conclusion, lanthanum chloride has been employed as a novel and efficient catalyst for the synthesis of benzimidazoles in good yields from *o*-phenylenediamine and a wide variety of aldehydes. All the reactions were carried out at room temperature while using the catalyst lanthanum chloride in 10 mol%. The reaction conditions were very mild, and the isolation of products was also very easy.

## Competing interests

The authors declare that they have no competing interests.

## Authors’ information

YV and SRK are research scholars, and PL is a professor at the Department of Chemistry, University College for Women, Koti Osmania University, Hyderabad 500095, India.
